# Optimization design and experiment of cam-elliptical gear combined vegetables curved surface labeling mechanism

**DOI:** 10.3389/frobt.2024.1431078

**Published:** 2024-12-13

**Authors:** Lei Zhang, Heng Zhou, Jianneng Chen, Junhua Tong, Yang Liu, Xiaowei Zhang

**Affiliations:** ^1^ School of Mechanical Engineering, Zhejiang Sci-Tech University, Hangzhou, Zhejiang, China; ^2^ Provincial Key Laboratory of Agricultural Intelligent Sensing and Robotics, Hangzhou, Zhejiang, China

**Keywords:** improved hypocycloid trajectory, cam-elliptical gear combined mechanism, entropy weight TOPSIS, quadratic optimization, NSGA - II

## Abstract

To address the problems of the labeling curved surfaces vegetable with long label, such as the label wrinkled and the easy detachment, a cam-elliptical gear combined labeling mechanism with an improved hypocycloid trajectory is proposed. Provide the process of the mechanism, and establish a kinematic model of the mechanism. In order to improve the motion performances of the cam-elliptical gear combined labeling mechanism and avoid labels damage, the NSGA-II algorithm is used to optimize the parameters of the mechanism, resulting in 80 sets of Pareto solutions. The entropy weight TOPSIS method is applied as a quadratic optimization to select an optimal solution from the 80 sets of Pareto solutions and obtain the optimized parameters of the mechanism. A comparative study is conducted with an elliptical-circular planetary gear mechanism using the hypocycloid trajectory. The results show that the improved mechanism reduces the maximum velocity by 7%, the maximum and minimum accelerations by 2% and 18%. After the quadratic optimization the distance error of the center point of suction cup and the labeling point is reduced from 1.3 mm to 0.12 mm, and the velocity during labeling and taking position is reduced from 0.10770 m s^−1^ to 0.0037 m s^−1^. The correctness of the proposed method is validated through simulation studies and experiments. This research provides a theoretical basis for the design and optimization of long label and curved surface labeling mechanism for vegetables.

## 1 Introduction

Vegetable production is becoming increasingly industrialized (Chen et al., 2023). Unman plant factories are evolving into high-tech facilities characterized by large-scale production and widespread use of technology (Zhang and Kacira, 2022; Jin et al., 2022a; Jin et al., 2022b) Labeling machines play an important role in the packaging of unmanned plant factories. Currently, the widely preferred labeling equipment includes blow-type labeling machines and bottle labeling machines, which can-not meet the long label labeling needs of curved surface vegetables.

Many scholars have conducted research on the labeling machines, such as Zhang et al. (2012) have designed a virtual prototype of a high-speed labeling mechanism based on Eon Studio software, but it has not been tested to verify its reliability. Cho and Bhushan (2016) studied the dam-age to labels caused by different labeling mechanism mate-rials, this provides a reference for the material selection of the labeling mechanism. Salamandra (2017) used light-sensitive sensors to locate the position of labels and improved labeling accuracy. In addition, the application of robots in labeling operations has also received attention (Wu et al., 2009a; Wu et al., 2009b; Wu et al., 2009c).

Sirkett et al. (2006), Sirkett et al. (2007) applied a spur gear planetary gear train with an hypocycloid trajectory to take the box, and verified its feasibility by finite element analysis method, but it did not establish mathematical modeling for a planetary gear train with an hypocycloid trajectory. Tong et al. (2018) designed an elliptical-circular gear planetary gear train mechanism, and established a kinematic model. It took a lot of time to obtain a set of optimal solutions by means of human-computer interaction, and proved the feasibility of the mechanism through comparative study. The lack of experiments verification does not prove whether it is feasible in real production. At the same time, the hypocycloid trajectory has been widely used in the mod-ern machinery industry (Mo et al., 2023; Toshinori and Ryota, 2023).

In terms of multi-objective optimization algorithms, Srinivas and Deb (Srinivas and Deb, 1995) proposed the NSGA. Cho and Lee (2017) focuses on the Multi-objective optimum design of tire structure using genetic algorithm. Xiong et al. (2018) used MOPSO and TOPSIS (Technique for Order Preference by Similarity to Ideal Solution) to optimize the weight of the car body. Leal-Naranjo et al. (2019) conducted a comparative study on the multi-objective optimization methods NGSA-Ⅱ, MOPSO and MOEA/D, it found that MOEA/D was more suitable for the dimensional synthesis of a spherical parallel manipulator. Palakonda and Kang (2023) proposed a Pre-DEMO on the basis of MODEA, and proved the correctness of the proposed algorithm through comparative study. Comparative study. Zhang et al. (2023) proposed a Grey Relation Analysis method for the secondary optimization of parameters after multi-objective optimization, which provided inspiration for avoiding human factor interference in the selection of optimal parameters in this paper.

In this study, the cam-elliptical gear combined mechanism is used to complete the curved surface labeling of vegetables. The specific research contents are as follows: establish a kinematics model of the cam-elliptic gear combined mechanism with an improved hypocycloidal trajectory, and give the labeling process. The parameters of the mechanism are optimized by multi-objective optimization method NSGA-Ⅱ and entropy weight TOPSIS method. According to the optimized parameters, a three-dimensional model of the cam-elliptical gear combined mechanism is established. The correctness of the mechanism is verified by theoretical analysis and simulation.

## 2 Materials and methods

### 2.1 Principle and process of the mechanism

**FIGURE 1:**
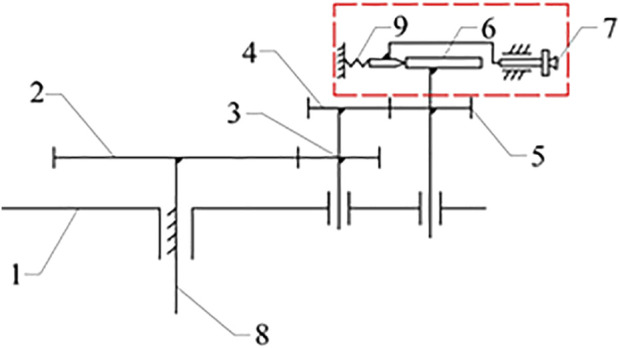
Cam-elliptical gear combined labeling mechanism: 1. rotating bracket 2. sun gear 3. spur idler gear 4. elliptical idler gear 5. elliptical planet gear 6. cam 7. labeling actuator 8. fixed shaft 9. spring.

The cam-elliptical gear combined labeling mechanism is shown in Figure 1. Its working principle is as follows: the rotating bracket one rotates around the fixed shaft 8. The sun gear 2 is fixed to the fixed shaft. The spur idler gear 3 and the elliptical idler gear 4 are fixed on the coaxial, and the elliptical idler gear is engaged with the elliptical planet gear 5. The cam 6 in the red line is coaxial with the elliptical planet gear, and the cam lever is placed behind the cam. The cam lever maintains contact with the cam through the action of spring 9. When the suction cup contacts the surface of the labeling object, the actuator 7 moves backward due to the action of the spring, thus achieving the purpose of flexible labeling.

**FIGURE 2:**
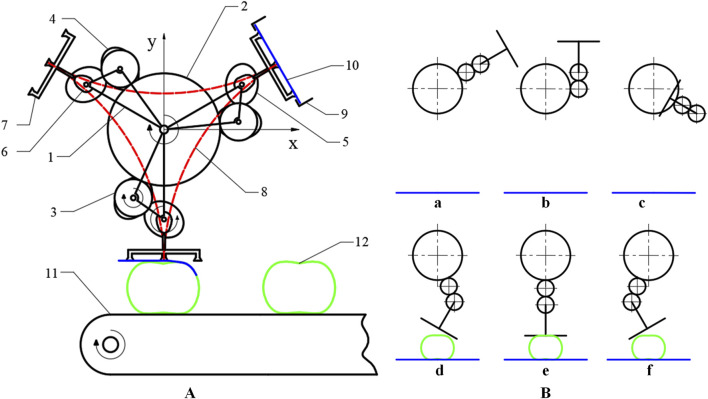
The process of cam-elliptical gear combined labeling mechanism **(A)**. 1. rotating bracket 2. sun gear 3. spur idler gear 4. elliptical idler gear 5. elliptical planet gear 6. cam 7. labeling actuator 8. Hypocycloid trajectory 9. label tray 10. label 11. conveyor 12. label object; **(B)** a-f is the different points in its travel.

The process of cam-elliptical gear combined labeling mechanism is shown in Figure 2A. During the labeling process, the moment when the suction cup on the labeling actuator makes contact with the label is considered the starting point of the labeling action. Firstly, the suction cup adsorbs the label, then the planetary gear train drives the actuator to follow the planetary gear rotation. When arriving at the labeling station, the suction cup affixes the label to the labeling object in the order of first right, then middle, and finally left. The operation is summarized in Figure 2B, which shows the different points in its travel.

### 2.2 The model of cam-elliptical gear combined labeling mechanism

#### 2.2.1 Mathematical model of cam-elliptical gear labeling mechanism

**FIGURE 3:**
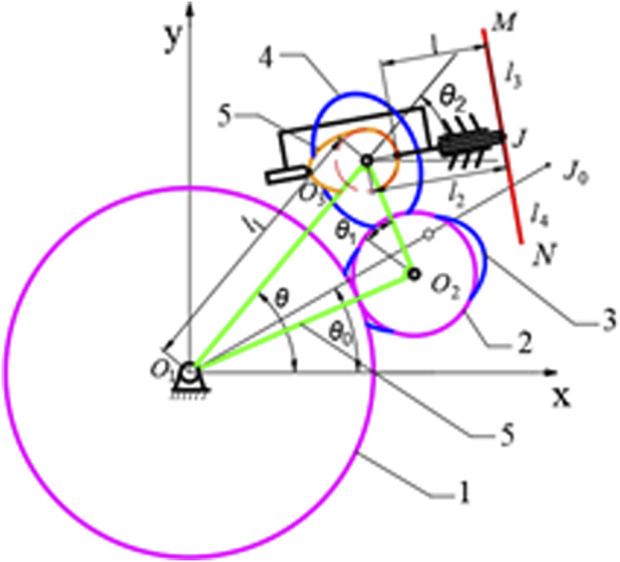
Structure diagram of C&E labeling mechanism 1. sun gear 2. circular idler gear 3. elliptical idler gear 4. Elliptical planetary gear 5. Cam 6. Rotating bracket.

The structure diagram of C&E labeling mechanism is shown in Figure 3. The symbols in the figure are defined as shows in Table 1.

**TABLE 1:** Parameter definition table.

Name	Definition
*O*_1_	Center of the sun gear
*O*_2_	Center of the idler gear
*O*_3_	Center of the elliptical planetary gear
*J*	Center point of the middle sucker of the labeling actuator
*M*	Center point of the right sucker of the labeling actuator
*N*	Center point of the left sucker of the labeling actuator
*J*_0_	Center point of the sucker after the end of the labeling actuator is displaced after *O*_1_ *O*_3_ rotation
*l*	Distance from the intersection of the concentric lines of *O*_3_ and *J* and the base circle of the cam to *J*
*l*_1_	Distance between the solar gear center *O*_1_ and the planetary gear center *O*_3_
*l*_2_	Distance between the planetary gear center *O*_3_ and point *J*
*l*_3_	Distance between the center point *J* of the middle sucker and the center point *M*
*l*_4_	Distance between the center point *J* of the middle sucker and the center point *N*
*θ*_0_	Initial angle between *O*_1_ *O*_3_ and the *x*-axis
*θ*_1_	The angle of the idler gear
*θ*_2_	The angle of the elliptical planetary gear
*σ*_1_	The angle between the short half axis of the elliptical idler gear and *O*_2_ *O*_3_

**FIGURE 4:**
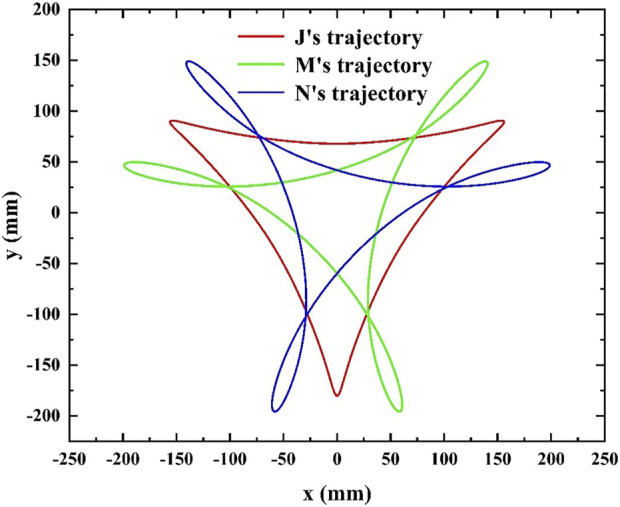
Hypocycloid trajectory.

Take *O*_*1*_ as the origin to establish a rectangular coordinate system as shown in Figure 4. The conditions for forming a hypocycloid trajectory, that is, the trajectory of the point of *J*, are as follows: the radius ratio of the sun gear, the idler gear and the planetary gear is 3:1:1; The initial angle between *O*_1_ *O*_3_ and the *x*-axis is *θ*_0_ equals *π*/6; The elliptical idler gear is the same size as the elliptical planetary gear. Therefore, when the rotating bracket rotates (*θ-θ*_0_), the elliptical planet gear will rotate 3(*θ-θ*_0_), that is, the suction cup will rotate from point *J* to *J*_0_. Establishes the displacement equation of the center point *J* of the elliptical-circular gear planetary gear train is in Equation 1:

$$\left\{\begin{array}{l} x=l_{1}\cos\theta +l_{2}\cos\left(\theta -\theta_{2}\right)\\ y=l_{1}\sin\theta +l_{2}\sin\left(\theta -\theta_{2}\right) \end{array}\right.$$ (1)

Displacement equation of the center point *M* of the right suction cup is in Equation 2:

$$\left\{\begin{array}{l} x=l_{1}\cos\theta +l_{2}\cos\left(\theta -\theta_{2}\right)-l_{3}\cos\left(\theta -\theta_{2}\right)\\ y=l_{1}\sin\theta +l_{2}\sin\left(\theta -\theta_{2}\right)+l_{3}\sin\left(\theta -\theta_{2}\right) \end{array}\right.$$ (2)

Displacement equation of the center point *N* of the left suction cup is in Equation 3:

$$\left\{\begin{array}{l} x=l_{1}\cos\theta +l_{2}\cos\left(\theta -\theta_{2}\right)+l_{4}\cos\left(\theta -\theta_{2}\right)\\ y=l_{1}\sin\theta +l_{2}\sin\left(\theta -\theta_{2}\right)-l_{4}\sin\left(\theta -\theta_{2}\right) \end{array}\right.$$ (3)

where *θ*_2_ is the rotation angle of the elliptical planetary gear relative to *O*_2_ *O*_3_; *l*_4_ is the distance between the point *J* and point *N*; *α* is the independent variable of the angle of the elliptical idler gear in Equation 4:

$$\theta_{2}=\int_{\sigma_{1}}^{\sigma_{1}+\theta_{1}}i_{21}d\alpha$$ (4)

where $\sigma_{1}$ is the angle between the minor axis of the elliptical idler gear and *O*_2_ *O*_3_; *i*_21_ is the transmission ratio of the elliptical gear in Equation 5:

$$i_{21}=\frac{1-e^{2}}{1+2e\cos\alpha +e^{2}}$$ (5)

where *e* is the eccentricity of the elliptical gear.

The trajectory equation of the point J of the cam-elliptical gear combined mechanism is in Equation 6:

$$\left\{\begin{array}{l} x=l_{1}\cos\theta +\left(l+s\right)\cos\left(\theta -\theta_{2}\right)\\ y=l_{1}\sin\theta +\left(l+s\right)\sin\left(\theta -\theta_{2}\right) \end{array}\right.$$ (6)

The distance *l*_2_ between the center of the planetary gear and the center point *J*, which is equal to *l* plus *s*, changes with the displacement of the cam push rod, and the change range is [*l*_2min_, *l*_2max_]. Where *l*_2min_ represents the distance between the cam center and the point *J* when the cam push rod is about to be pushed travel. *L*_2max_ represents the distance when the cam push rod is about to make a return travel.

Where s is the travel of the cam push rod. This paper studies the trajectory of sine acceleration curve cam, and the motion equations are in Equation 7 (Podgornyj et al., 2019):

$$s=\left\{\begin{array}{cc} h\left[1-\cos\left(\pi \delta /\delta_{0}\right)\right]/2 & ,0\leq \delta <\pi \\ h\left[1-\cos\left(\pi \delta /\delta_{0}\right)\right]/2 & ,\pi \leq \delta \leq 2\pi \end{array}\right.$$ (7)

where *s* is sine acceleration and constant velocity; *δ* is the cam motion angle, *δ* is equal to *θ*_2_; *δ*_0_ is the cam motion angle, 0 ≤ *δ*_0_ ≤ 180° is the return travel, 180° ≤ *δ*_0_ ≤ 360° is the push travel; *h* is the stroke of the push rod.

#### 2.2.2 Kinematics model of cam-elliptical gear combined labeling mechanism

The motion performances of the labeled mechanism are related to the rotation angle of the rotating bracket. The rotation velocity is set at 60 rad min^−1^ and the theoretical production efficiency is 180 pcs min^−1^. The rotation angle of the rotating bracket is *θ* equals 2*πt*, and the velocity equation of the point *J* is in Equation 8:

$$\left\{\begin{array}{l} v_{x}=-2\pi l_{1}\sin2\pi t-\left(l+s\right)\sin\left(2\pi t-\theta_{2}\right)\left(2\pi +{\overset{\cdot }{\theta }}_{2}\right)+\overset{\cdot }{s}\cos\left(2\pi t-\theta_{2}\right)\\ v_{y}=2\pi l_{1}\cos2\pi t+\left(l+s\right)\cos\left(2\pi t-\theta_{2}\right)\left(2\pi +{\overset{\cdot }{\theta }}_{2}\right)+\overset{\cdot }{s}\sin\left(2\pi t-\theta_{2}\right) \end{array}\right.$$ (8)

where $\dot{s}$ is the derivative of *s*; $\theta_{2}$ is the rotation angle of the elliptical planetary gear relative to *O*_2_ *O*_3_ in Equation 9; ${\overset{\cdot }{\theta }}_{2}$ is the angular velocity of the elliptical planetary gear is in Equation 10:

$$\theta_{2}=\int_{0}^{2\pi t-\frac{\pi }{2}}\frac{-\left(e^{2}-1\right)}{e^{2}+2e\cos\alpha +1}d\alpha$$ (9)

$$\overset{\cdot }{\theta_{2}}=\frac{2\pi \left(e^{2}-1\right)}{e^{2}+2e\cos\left(2\pi t-\frac{\pi }{2}\right)+1}$$ (10)

Velocity is in Equation 11:

$$v=\sqrt{{v_{x}}^{2}+{v_{y}}^{2}}$$ (11)

### 2.3 Parameters optimization based on NSGA-II and entropy weight TOPSIS

An optimization method of cam-elliptical gear labeling mechanism based on NSGA-II and entropy weight TOPSIS is proposed. The Pareto Frontier solution set is obtained by NSGA-Ⅱ after the first optimization (Kumar et al., 2009; Choi et al., 2021), and then the entropy weight TOPSIS method is used as a quadratic optimization to obtain the optimal solution of the mechanism parameters. The specific steps of the algorithm are as follows.

① Establishment of objective function:

Set the label starting point *X*_*r*_: (55, −195), the middle point *X*_*m*_: (0, −180), the label ending point *X*_*l*_: (−55, −195), and the arc length from the starting point to the ending point is 135 mm. To ensure that the label on the point *M* can be successfully attached to the labeling starting point *X*_*r*_, the distance between the farthest point of the trajectory of the point *M* and the labeling starting point is optimized as one of the objective functions. This objective function can be expressed in Equation 12:

$$f_{1}\left(x\right)=\min\left(\sqrt{{\left(x_{\max}-x_{r}\right)}^{2}+{\left(y_{\max}-y_{r}\right)}^{2}}\right)$$ (12)

where *x*_max_, *y*_max_ are the values of coordinate *x* and *y* when the value of *f*_0_ (*x*,*y*) is the largest, which can be expressed in Equation 13:

$$\max f_{0}\left(x,y\right)=\sqrt{x_{\max}^{2}+y_{\max}^{2}},x>0,y<0$$ (13)

The starting point and ending point of labeling are symmetric about the *y*-axis. The left and right sides labeling trajectories are also symmetric about the *y*-axis. Thus, the right side labeling can complete the labeling action, and the left side can also complete the labeling action. Therefore, in the process of optimization, only the distance between the point *M* and the labeling starting point *X*_*r*_ is taken as the objective function.

Because there is a short touch between the label and the label’s plate when taking the label, the label is easy to fall. If the velocity is too fast, the label will be damaged. Therefore, the velocity of the taking station and the labeling station is as small as possible, so the minimum value of the velocity is used as another optimization objective in Equation 14:

$$f_{2}\left(x\right)=\min\left(v_{\min}\right)$$ (14)

where *v*_min_ is the minimum value of the velocity, that is, the value of the velocity when the angle is 0, 2*π*/3, 4*π*/3.

Then the optimization model can be expressed in Equation 15:

$$\min F\left(X\right)={\left[f_{1}\left(x\right),f_{2}\left(x\right)\right]}^{T}$$ (15)

② Establishment of target variables in Equation 16:

$$x=\left[l_{1}\right.\left.\;l_{2}c\;a\;l_{3}\right]$$ (16)

③ Establishment of constraint conditions:

To ensure smooth operation of the elliptical gear without pulsation (Bair, 2004) [(*a*+*c*)/(*a*-*c*)]^2^ ≤ 5; To ensure that the mechanism can complete the hypocycloidal trajectory, the relationship between *l*_1_ and *l*_2max_ is -*l*_1_+2*l*_2max_ ≤ −1; To ensure that the middle suction cup can reach the labeling point, *l*_1_ + (*l*_2max_ + s) = 180; In order to get the proper distance between the middle sucker and the right sucker, -*l*_2max_ + *l*_3_ ≤ 20. The constraint equation can be established in Equation 17:

$$s.t.\;\left\{\begin{array}{l} {\left(\frac{a+c}{a-c}\right)}^{2}\leq 5\\ -l_{1}+2l_{2}\leq 1\\ l_{1}+l_{2}=172\\ -l_{2}+l_{3}\leq 20 \end{array}\right.$$ (17)

According to the parameters of the labeled object, the value range of each variable is considered comprehensively in Equation 18:

$$s.t.\;\left\{\begin{array}{l} 100\leq l_{1}\leq 140\\ 50\leq l_{2}\leq 70\\ 1\leq c\leq 20\\ 20\leq a\leq 50\\ 35\leq l_{3}\leq 85 \end{array}\right.$$ (18)

④ Establishment of the original decision matrix:

The performance index of Pareto Frontier solution obtained by NSGA-Ⅱ optimization is transformed into the original decision matrix in TOPSIS method in Equation 19:

$$X=\left(\begin{array}{cccc} x_{11} & x_{12} & \cdots & x_{1n}\\ x_{21} & x_{22} & \cdots & x_{2n}\\ \vdots & \vdots & \ddots & \vdots \\ x_{m1} & x_{m2} & \cdots & x_{mn} \end{array}\right)$$ (19)

where *X* is the original decision matrix, *x*_*ij*_ represents the *j* index value of the *i* scheme, *i* = 1,2, … *m*, *j* = 1,2, … *n*, *x*_*ij*_ in this study is the Pareto solutions obtained by NSGA-II algorithm, *m* = 80, *n* = 2.

⑤ Establishment of regular matrix:

In order to eliminate the effects of different dimensions, each element of the original matrix needs to be normalized in Equation 20:

$$z_{ij}=a_{ij}/\sum_{i=1}^{m}a_{ij}$$ (20)

The regular matrix can be obtained in Equation 21:

$$Z_{ij}=\left(\begin{array}{cccc} z_{11} & z_{12} & \cdots & z_{1n}\\ z_{21} & z_{22} & \cdots & z_{2n}\\ \vdots & \vdots & \cdots & \vdots \\ z_{m1} & z_{m2} & \cdots & z_{mn} \end{array}\right)$$ (21)

⑥ Determine entropy weight and information entropy:

The information entropy and weight coefficient of each performance index of cam-elliptical gear combined labeling machine were calculated, respectively. The calculation formula of information entropy is in Equation 22:

$$H_{j}=-\frac{1}{\ln\left(m\right)}\sum_{i=1}^{m}z_{ij}\ln\left(z_{ij}\right)$$ (22)

The calculation formula of entropy weight coefficient is in Equation 23:

$$\omega_{j}=\left(1-H_{j}\right)/\left(m-\sum_{j=1}^{m}H_{j}\right)$$ (23)

⑦ Calculate the normalized score:

Determine positive and negative ideal solutions in Equation 24:

$$\left\{\begin{array}{l} Z^{+}=\left(Z_{1}^{+},Z_{2}^{+},\cdots ,Z_{m}^{+}\right)\\ =\left(\max\left(z_{11},z_{21},\cdots ,z_{n1}\right),\max\left(z_{12},z_{22},\cdots ,z_{n2}\right),\cdots ,\max\left(z_{1m},z_{2m},\cdots ,z_{nm}\right)\right)\\ Z^{-}=\left(Z_{1}^{-},Z_{2}^{-},\cdots ,Z_{m}^{-}\right)\\ =\left(\min\left(z_{11},z_{21},\cdots ,z_{n1}\right),\min\left(z_{12},z_{22},\cdots ,z_{n2}\right),\cdots ,\min\left(z_{1m},z_{2m},\cdots ,z_{nm}\right)\right) \end{array}\right.$$ (24)

where Z^+^ and Z^−^ represent positive ideal solution set and negative ideal solution set, respectively; $Z_{m}^{+}$ represents the largest number in each column and $Z_{m}^{-}$ represents the smallest number in each column.

TOPSIS method uses the Euclidean distance between each index and the positive and negative ideal solution to select its advantages and disadvantages.The corresponding calculation formula is in Equation 25:

$$\left\{\begin{array}{l} D_{i}^{+}=\sqrt{\sum_{j=1}^{m}\omega_{j}{\left(Z_{ij}-Z_{j}^{+}\right)}^{2}}\\ D_{i}^{-}=\sqrt{\sum_{j=1}^{m}\omega_{j}{\left(Z_{ij}-Z_{j}^{-}\right)}^{2}} \end{array}\right.$$ (25)

Calculate the normalized score in Equation 26:

$$S=\frac{D_{i}^{-}}{D_{i}^{+}+D_{i}^{-}}$$ (26)

where 0 ≤ *S* ≤ 1, the closer the value of *S* is to 1, and the larger the value of $D_{i}^{+}$ , it indicates that the evaluation effect of the proposed solution is superior (Qu et al., 2023).

## 3 Results

### 3.1 Optimization results of cam-elliptical gear combined labeling mechanism

#### 3.1.1 The results of the comparative study

**FIGURE 5:**
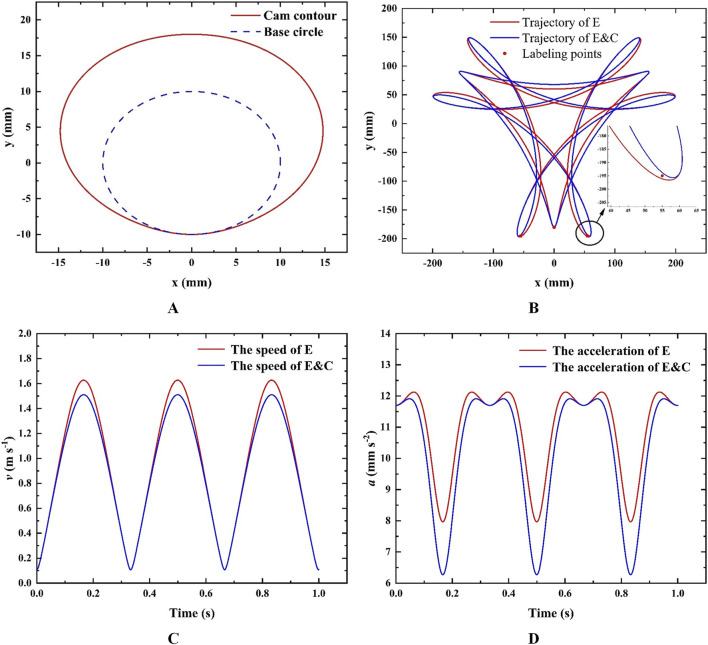
**(A)** Cam profile; **(B)** Track curve; **(C)** Velocity comparison; **(D)** Acceleration comparison.

The parameters of the cam-elliptical gear combined labeling mechanism are initially selected: take *l*_1_ as 120, *l* as 52, *l*_3_ as 60, *c* as one and *a* as 20. Take the radius of the base circle of the cam *r*_0_ as 10, take stroke *s* as 8. By applying the parameters into Equation 7, the contour of the cam can be obtained, resulting in the visualization shown in Figure 5A. Similar comparative analysis is made on the cam-elliptical gear combined labeling mechanism. Perform a comparative analysis of the cam-elliptical gear combined labeling mechanism (For ease of narration, the legend in Figure 5 uses E&C instead of the cam-elliptical gear combined labeling mechanism), and obtain the hypocycloid trajectory, velocity curve, and acceleration curve for the elliptical-circular gear planetary gear labeling mechanism (For ease of narration, the legend in Figure 5 uses E instead of the elliptical-circular gear planetary gear labeling mechanism). Compare it with the cam-elliptical gear combined label applicator mechanism as shown in Figure 5. The distance error of the point *M* and the point *X*_*r*_, as well as the maximum velocity, maximum acceleration and minimum acceleration of the point *J* trajectory are compared, as shown in Table 2. It can be seen that the distance error is reduced from 2.3 mm to 1.3 mm, the maximum velocity is reduced by 7%, and the minimum acceleration is reduced by 18%. Maximum acceleration reduced by 2%.

**TABLE 2:** Comparison of motion performances after adding cam.

	Distance error (mm)	Maximum velocity (m s^−1^)	Maximum acceleration (mm s^−2^)	Minimum acceleration (mm s^−2^)
E labeling mechanism	2.3	1.668	12,660	8,222
E&C labeling mechanism	1.3	1.552	12,384	7,528
difference Value	1	0.116	276	1,694
Percentage	43%	7%	2%	18%

The results show that the cam-elliptical gear labeling mechanism has better motion performances. Although the motion performances are improved, it can be seen from Figure 5C and Table 2 that there is a certain distance error of the maximum point *M* trajectory and the point *X*_*r*_. And there is still a certain velocity in the taking and labeling station, which affects the marking and labeling effect. Therefore, it is necessary to optimize the parameters of the mechanism.

#### 3.1.2 Result of the optimization

**FIGURE 6:**
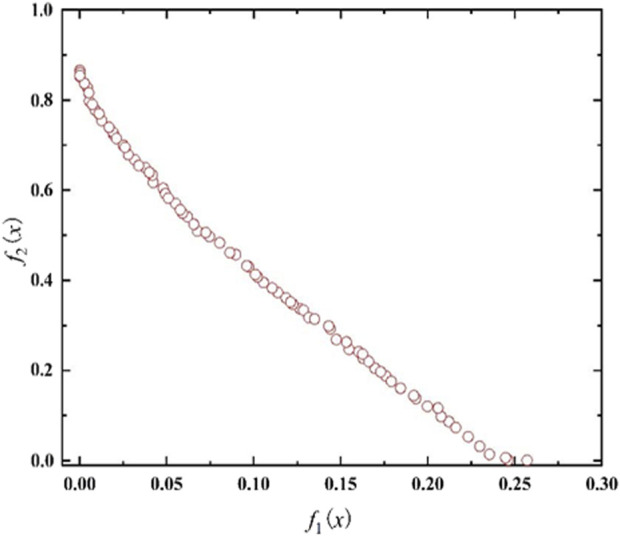
Pareto sets.

Set the population number be 200, the maximum genetic algebra be 800, and the crossover probability be 0.4. The optimal Frontier of Pareto is shown in Figure 6. It can be seen that eighty sets of Pareto solutions are smooth as a whole, and the solution sets is evenly distributed, it shows that the optimization results are good. The optimization results indicate that there are mutual constraints between the objectives and the optimal solution cannot be obtained at the same time.

**FIGURE 7:**
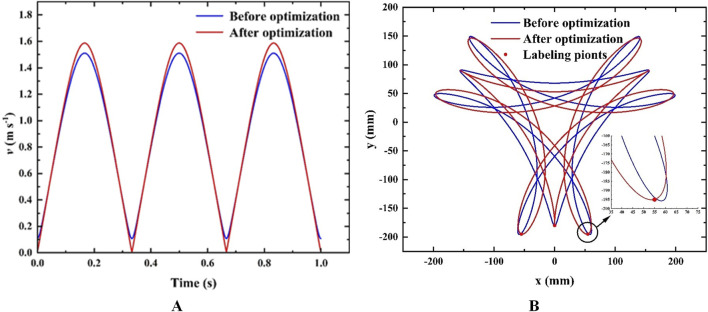
**(A)** Optimized trajectory comparison; **(B)** Optimized velocity comparison.

Therefore, the entropy weight TOPSIS method is used as a quadratic optimization to evaluate the 80 of solutions and find an optimal solution. The eighty sets of Pareto solutions obtained from calculations are taken as the candidate decision solutions. The information entropy and entropy weight of the minimum distance $f_{1}\left(x\right)$ and minimum velocity $f_{2}\left(x\right)$ are calculated and shown in Table 3. The relative closeness between each scheme and the ideal solution is shown in Table 4. At the end, the 67th set of solutions is selected as the optimal solution with the parameters: take *l*_1_ as 114.23, *l* as 57.77, *c* as 1.06, *a* as 22.75 and *l*_3_ as 60.04. Comparing the trajectory of the point *J*, the point *N*, and the point *M* before and after optimization, and the velocity of *J*, as shown in Figures 7A, B.

**TABLE 3:** Information entropy and entropy weight of each evaluation index.

Evaluation index	Information entropy	Entropy weight
$f_{1}\left(x\right)$	0.968	39.247
$f_{2}\left(x\right)$	0.951	60.753

**TABLE 4:** The relative closeness between each scheme and the ideal solution.

Ranking	$D_{i}^{+}$	$D_{i}^{-}$	*S*	Projects
1	0.464	0.650	0.584	67
2	0.472	0.660	0.583	40
⋮				
79	0.775	0.626	0.447	2
80	0.779	0.626	0.445	1

The distance error of the maximum point of the *M* trajectory and the point *X*_*r*_ before and after optimization, and the velocity of the point *J* in labeling and taking station, are shown in Table 5. The results show that the cam-elliptical gear combined labeling mechanism has better motion performances while achieving the labeling point.

**TABLE 5:** Comparison of optimization results.

	Distance error (mm)	Labeling velocity (m s^−1^)
Before optimization	1.30	0.10770
After optimization	0.12	0.0037

### 3.2 Results of the simulation and analysis

**FIGURE 8:**
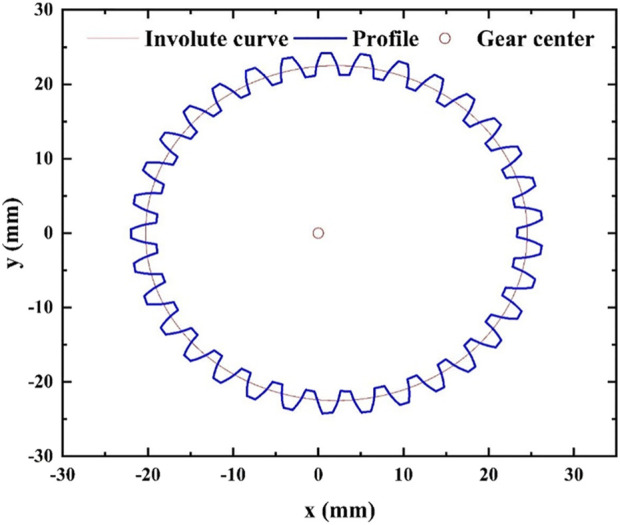
Pitch curve and tooth profile of elliptical gear.

**FIGURE 9:**
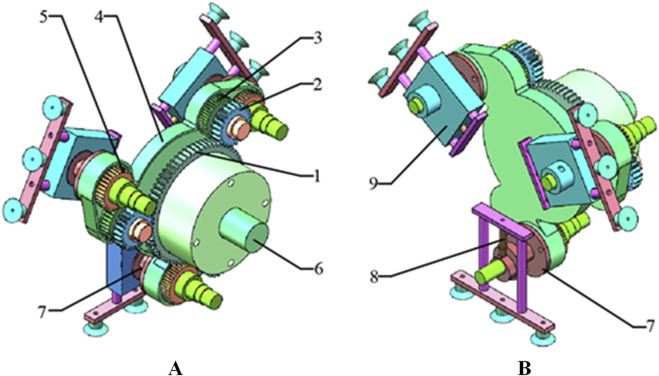
Three-dimensional model of cam-elliptical gear combined labeling mechanism: **(A)** The back of mechanism, 1. sun gear 2. idler gear 3. elliptical idler gear 4. rotating bracket 5. elliptical planetary gear 6. fixed shaft 7. cam; **(B)** The front of mechanism, 8. cam lever, 9. sliding sleeve.

Based on the optimized parameters, the tooth profile and pitch curve of the elliptical gear are obtained (Bair, 2002; Ye et al., 2019; Marafona et al., 2024), as shown in Figure 8. Established the three-dimensional model of cam-elliptical gear combined labeling mechanism as shown in Figure 9. It can see that the main body of the mechanism is composed of elliptical and circular gear planetary gear train, in a 120° symmetrical arrangement, where the idler gear and planetary gear are installed on the rotating bracket, the labeling actuator is fixed on the planetary gear shaft, and the suction cup is installed on the left end, middle end and right end of the labeling actuator. When the rotating bracket is rotated, the idler gear is driven to rotate around the sun gear, and the idler gear transmits the power to the planetary gear, and the planetary gear drives the labeling actuator to rotate.

**FIGURE 10:**
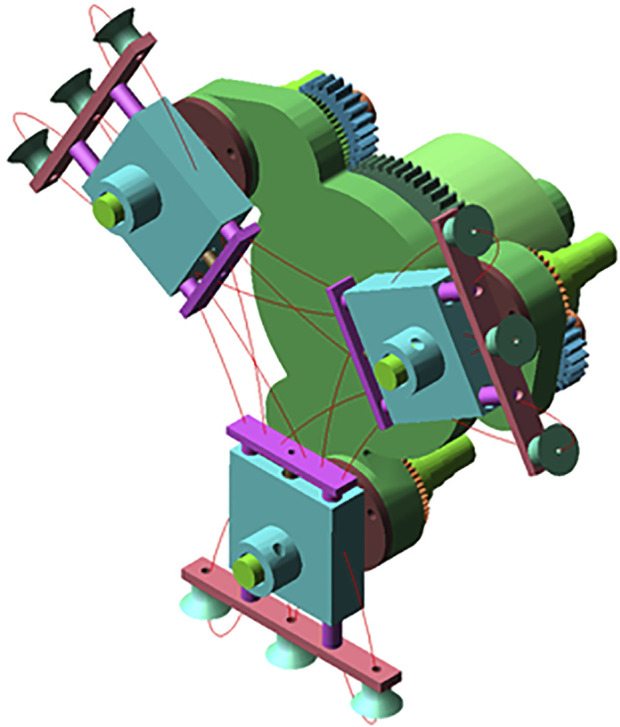
Simulation trajectory.

**FIGURE 11:**
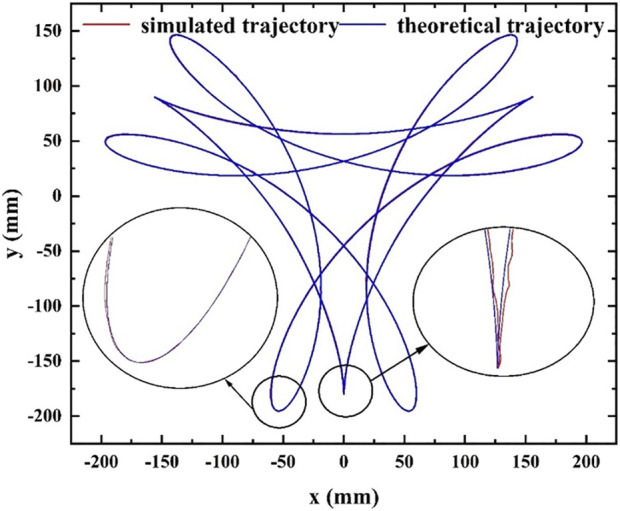
Comparison between simulation trajectory and theoretical trajectory.

To conduct simulation analysis and obtain the simulation trajectory of the cam-elliptical gear combined labeling mechanism, Adams software is utilized. The results of the simulation are depicted in Figure 10. By comparing the theoretical trajectory and the simulation trajectory in Figure 11, it can be seen that the theoretical trajectory and the simulation trajectory are basically the same. The simulation trajectory will fluctuate during the taking and labeling stations, because the gear will collide during operation and cause the fluctuation phenomenon.

### 3.3 Experiment of cam-elliptical gear labeling mechanism

#### 3.3.1 Construction of cam-elliptical gear combined labeling machine

**FIGURE 12:**
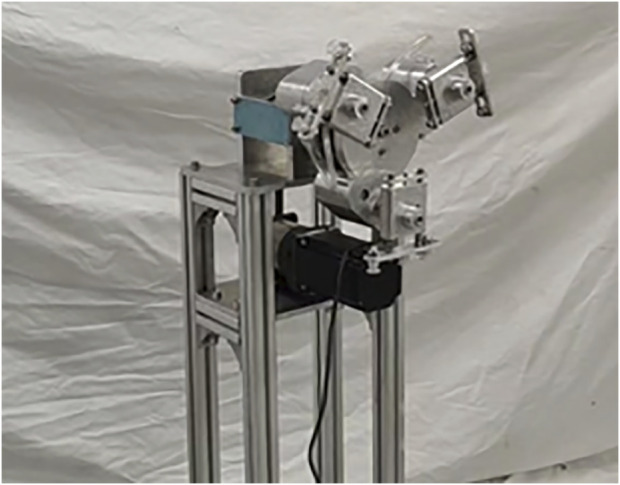
Cam-elliptical gear combination vegetables curved surface labeling mechanism.

The two-dimensional drawings of each part of the designed packaging labeling machine are exported by SolidWorks software, and processed according to the drawn two-dimensional drawings. The structure of the whole machine after assembly is shown in Figure 12.

#### 3.3.2 The track verification

**FIGURE 13:**
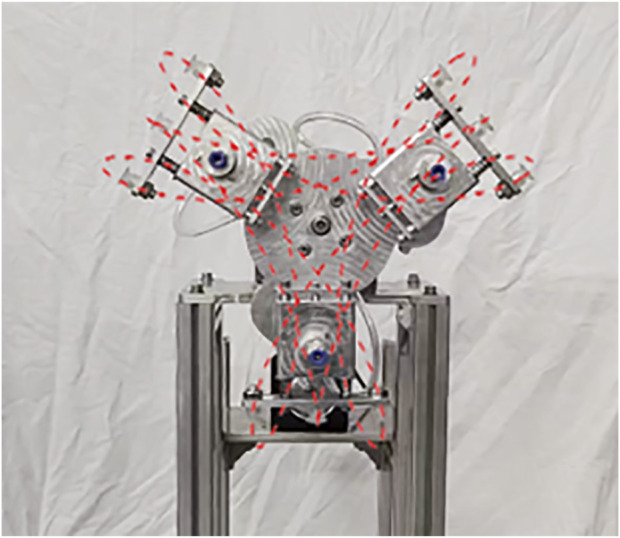
Actual trajectory.

Firstly, the test device of the labeling machine is placed in an indoor environment with sufficient light, and the prototype is controlled by the motor controller to achieve the appropriate speed, and the running labeling machine is photographed by the photographic equipment. Then, the video of a running cycle is imported into Adobe Premiere Pro video analysis software, and the running trajectory of *J* point from mark-taking to labeling to mark-taking process is analyzed frame by frame. Finally, the overall motion trajectory of the point *J* of the cam-elliptic gear wrapper surface labeling mechanism is obtained, as shown in Figure 13.

Comparing the test trajectory with the theoretical trajectory calculated in Chapter 2.2 with the simulation trajectory obtained by Adams software in Chapter 3.2, the following results are obtained.

(1) Comparing the actual trajectory with the other two trajectories, it can be seen that there is a certain error between the actual trajectory and the other two trajectories in the middle arc segment, but the overall shape and height are not much different. It can be seen from the figure that there is a certain fluctuation in the actual trajectory during the marking and labeling sections. The reason for the fluctuation is that the machining accuracy of the elliptical gear is not enough, and the gap between the teeth does not meet the requirements. In addition, the machining error of other parts, the vibration generated during the installation and operation of the equipment are also the causes of the trajectory formation error.
(2) From the point of view of the running speed of the actual trajectory, the rotation of the cam-elliptic gear packaging dish surface labeling machine prototype. The rotation speed can reach 20 min/rad, and the running speed can reach 60pcs/min due to three sets of labeling actuators.

In summary, the actual trajectory of the cam-elliptical gear packaging dish surface labeling mechanism meets the requirements.

## 4 Conclusion

(1) To address the curved surface labeling problem of packaged vegetables in unmanned plant factories, the tricuspid hypocycloid was identified as the labeling trajectory, and an improved tricuspid hypocycloid trajectory was proposed, and the improved tricuspid hypocycloid parametric equation was established.
(2) The mathematical models of cam-elliptical gear combined mechanism and elliptical-circular gear mechanism are established, respectively. The results show that the motion performances of the cam-elliptical gear combined mechanism are improved during the labeling process. Taking the main rotational velocity of 60 rad min^−1^ as an example, the maximum velocity of the point M of the cam-elliptical gear combined mechanism during the labeling process is reduced by 7%, and the maximum and minimum acceleration are reduced by 2% and 18%. The data show that the cam-elliptical gear combined mechanism has better motion performances.
(3) Firstly, NSGA algorithm is used to optimize the cam-elliptical gear combined mechanism, and 80 sets of Pareto solutions are obtained. Then entropy weight TOPSIS method is used as a quadratic optimization to select a set of optimal solutions. Secondly, the 67th set of solutions is selected as the optimal solution. The distance error of the maximum point of the point *M* and the starting point *X*_*r*_ is reduced from 1.30 mm to 0.12 mm, and the velocity at the labeling and taking station is reduced from 0.10770 m s^−1^ to 0.0037 m s^−1^. Finally, the correctness of the designed mechanism is verified by experiments.

## Data Availability

The original contributions presented in the study are included in the article/supplementary material, further inquiries can be directed to the corresponding author.
