# Supplementary material for: Optimization design and experiment of cam-elliptical gear combined vegetables curved surface labeling mechanism
Source: Front Robot AI. 2024 Dec 13;11:1431078. doi: 10.3389/frobt.2024.1431078 (PMC11681500; doi:10.3389/frobt.2024.1431078)
Supplement: Supplementary file 1 [file Supplementaryfile1.docx]

Supplementary Material

# Supplementary Figures and Tables

**Supplementary Table 1.** Parameter definition table

| Name | Definition |
| --- | --- |
| *O*_1_ | Center of the sun gear |
| *O*_2_ | Center of the idler gear |
| *O*_3_ | Center of the elliptical planetary gear |
| *J* | Center point of the middle sucker of the labeling actuator |
| *M* | Center point of the right sucker of the labeling actuator |
| *N* | Center point of the left sucker of the labeling actuator |
| *J*_0_ | Center point of the sucker after the end of the labeling actuator is displaced after *O*_1_*O*_3_ rotation |
| *l* | Distance from the intersection of the concentric lines of *O*_3_ and *J* and the base circle of the cam to *J*. |
| *l*_1_ | Distance between the solar gear center *O*_1_ and the planetary gear center *O*_3_ |
| *l*_2_ | Distance between the planetary gear center *O*_3_ and point *J* |
| *l*_3_ | Distance between the center point *J* of the middle sucker and the center point *M* |
| *l*_4_ | Distance between the center point *J* of the middle sucker and the center point *N* |
| *θ*_0_ | Initial angle between *O*_1_*O*_3_ and the *x*-axis |
| *θ*_1_ | The angle of the idler gear |
| *θ*_2_ | The angle of the elliptical planetary gear |
| *σ*_1_ | The angle between the short half axis of the elliptical idler gear and *O*_2_*O*_3_ |

**Supplementary Table 2.** Comparison of motion performances after adding cam

|  | distance error (mm) | Maximum velocity  (mm s^-1^) | Maximum acceleration  (mm s^-2^) | Minimum acceleration  (mm s^-2^) |
| --- | --- | --- | --- | --- |
| E labeling  mechanism | 2.3 | 1668 | 12660 | 8222 |
| E&C labeling  mechanism | 1.3 | 1552 | 12384 | 7528 |
| difference Value | 1 | 116 | 276 | 1694 |
| Percentage | 43% | 7% | 2% | 18% |

**Supplementary Table 3.** Information entropy and entropy weight of each evaluation index

| Evaluation index | Information entropy | Entropy weight |
| --- | --- | --- |
| $f_{1}(x)$ | 0.968 | 39.247 |
| $f_{2}(x)$ | 0.951 | 60.753 |

**Supplementary Table 4.** The relative closeness between each scheme and the ideal solution

| Ranking | $D_{i}^{+}$ | $D_{i}^{-}$ | *S* | Projects |
| --- | --- | --- | --- | --- |
| 1 | 0.464 | 0.650 | 0.584 | 67 |
| 2 | 0.472 | 0.660 | 0.583 | 40 |
|  |  |  |  |  |
| 79 | 0.775 | 0.626 | 0.447 | 2 |
| 80 | 0.779 | 0.626 | 0.445 | 1 |

**Supplementary Table 5.** Comparison of optimization results

|  | Distance error  (mm) | Labeling velocity  (m s^-1^) |
| --- | --- | --- |
| Before optimization | 1.30 | 0.10770 |
| After optimization | 0.12 | 0.0037 |

# Supplementary Figures


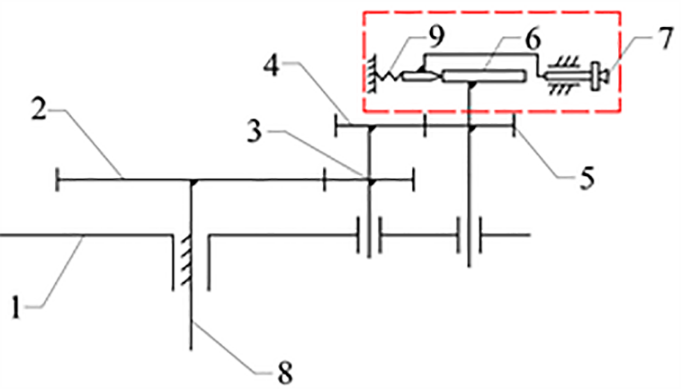


Supplementary Figure 1. Cam-elliptical gear combined labeling mechanism: 1.rotating bracket 2.sun gear 3.spur idler gear 4.elliptical idler gear 5.elliptical planet gear 6.cam 7.labeling actuator 8.fixed shaft 9.spring.


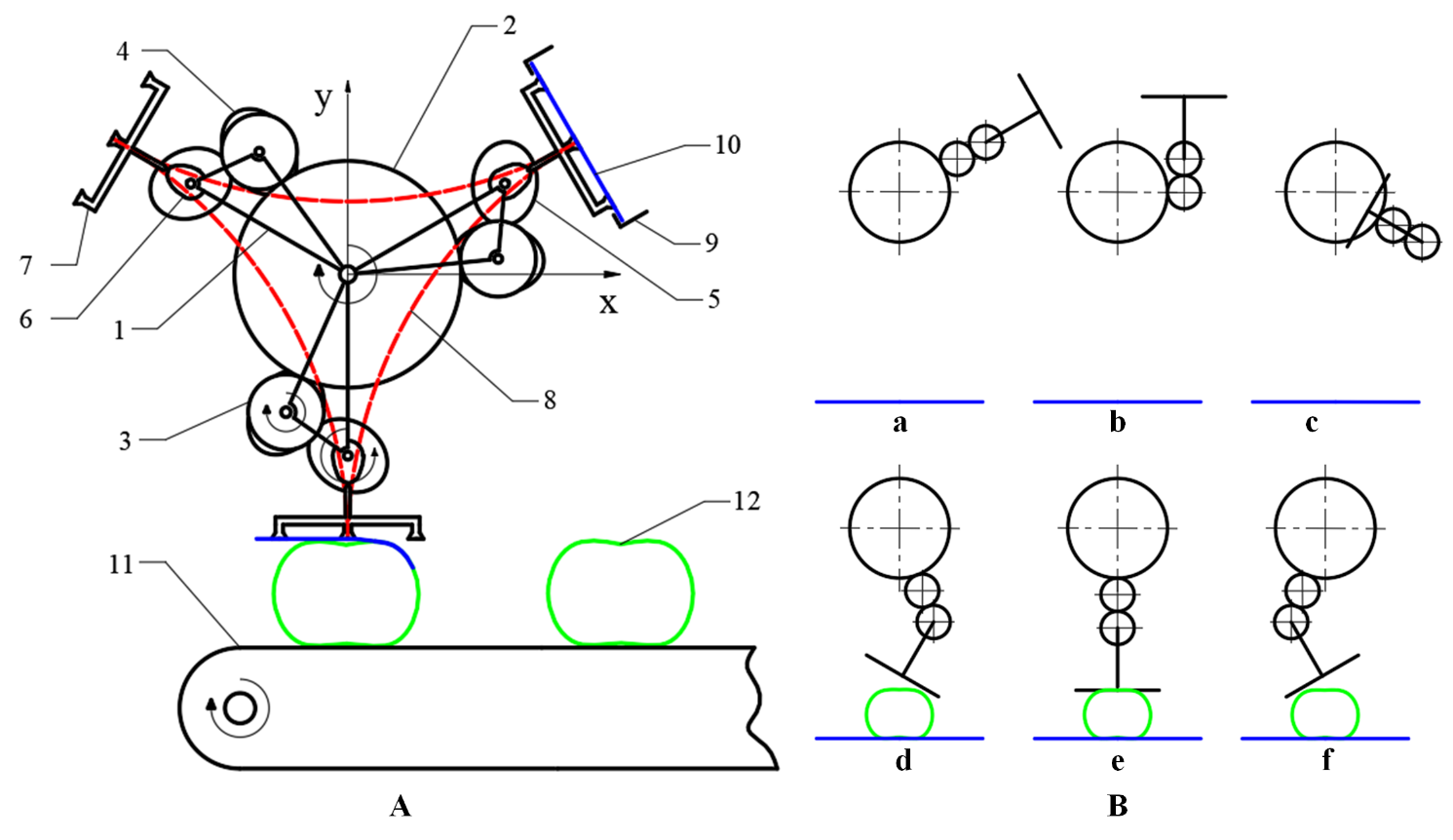


Supplementary Figure 2. The work flow of cam-elliptical gear combined labeling mechanism: (A) 1.rotating bracket 2.sun gear 3.spur idler gear 4.elliptical idler gear 5.elliptical planet gear 6.cam 7.labeling actuator 8. hypocycloid trajectory 9.label tray 10.label 11.conveyor 12.label object; (B) a-f is the gear train at different points in its travel.


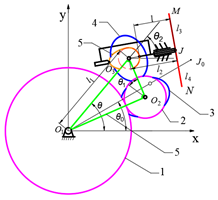


Supplementary Figure 3. Structure diagram of C&E labeling mechanism 1.sun gear 2.circular idler gear 3.elliptical idler gear 4. elliptical planetary gear 5. cam 6. rotating bracket


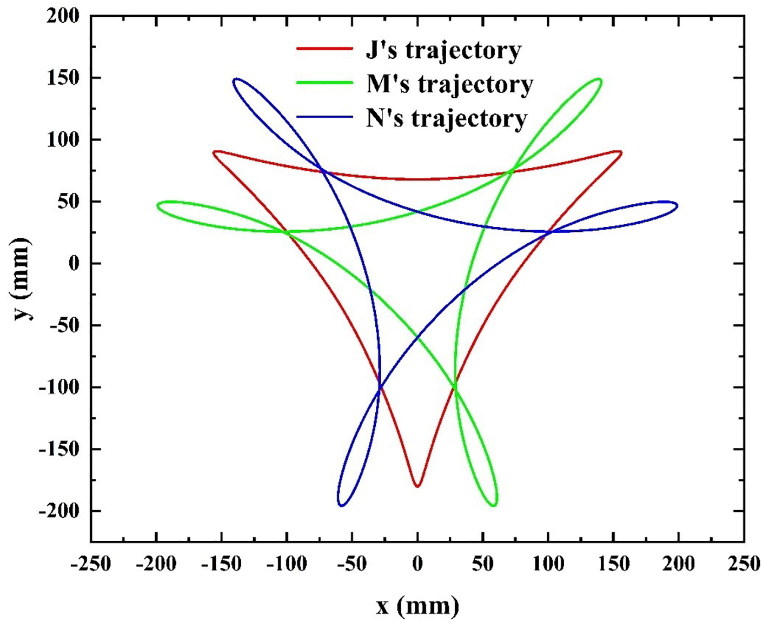


Supplementary Figure 4. Hypocycloid trajectory


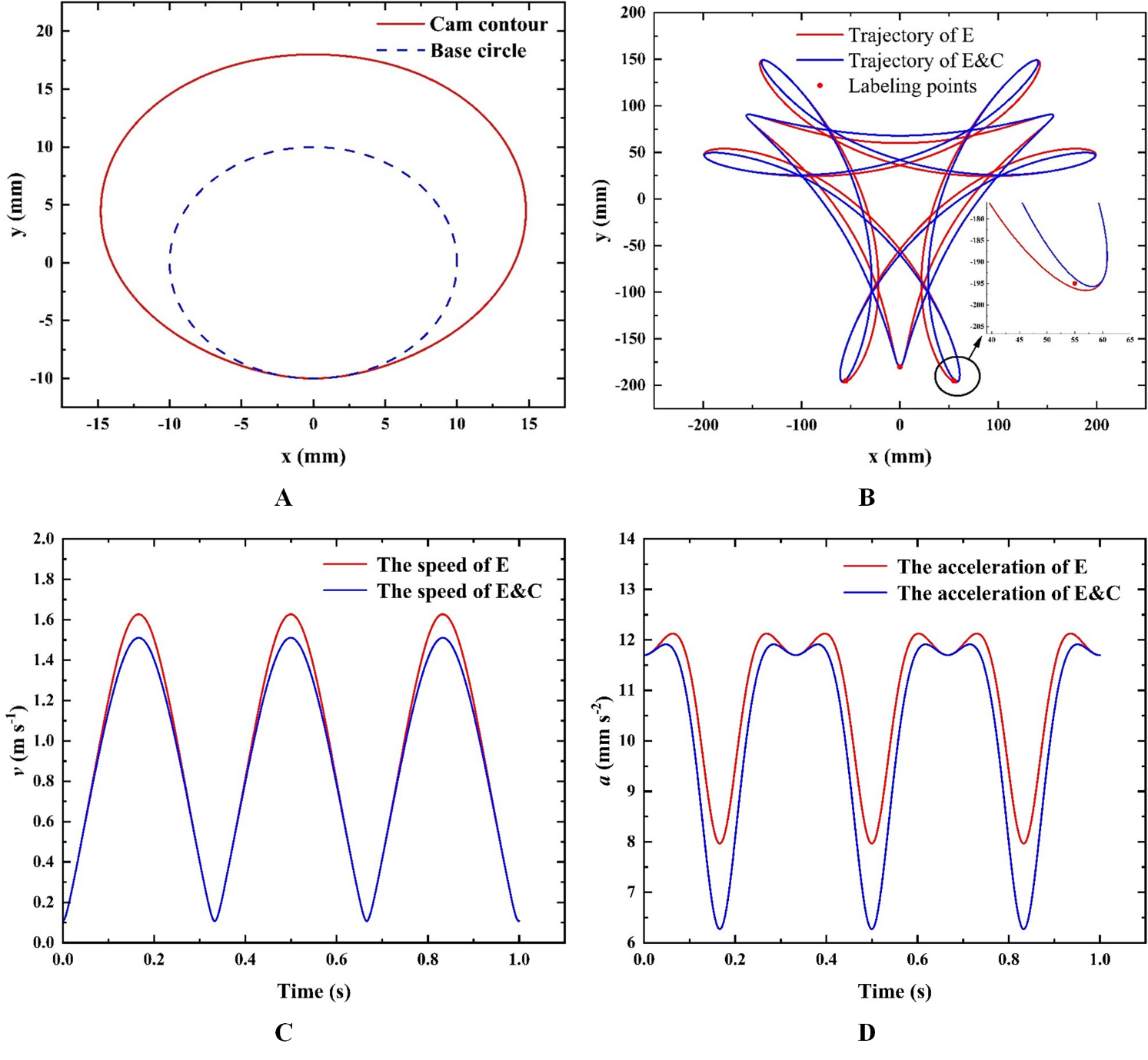


Supplementary Figure 5. (A) Cam profile; (B) Track curve; (C) Velocity comparison; (D) Acceleration comparison


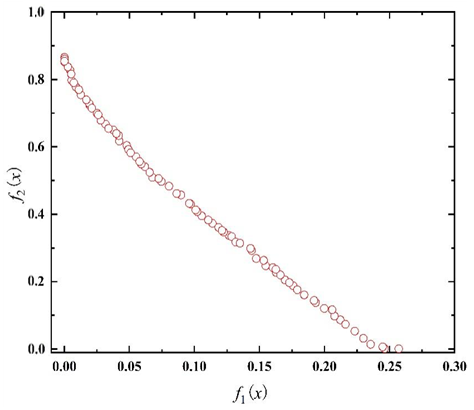


Supplementary Figure 6. Pareto sets


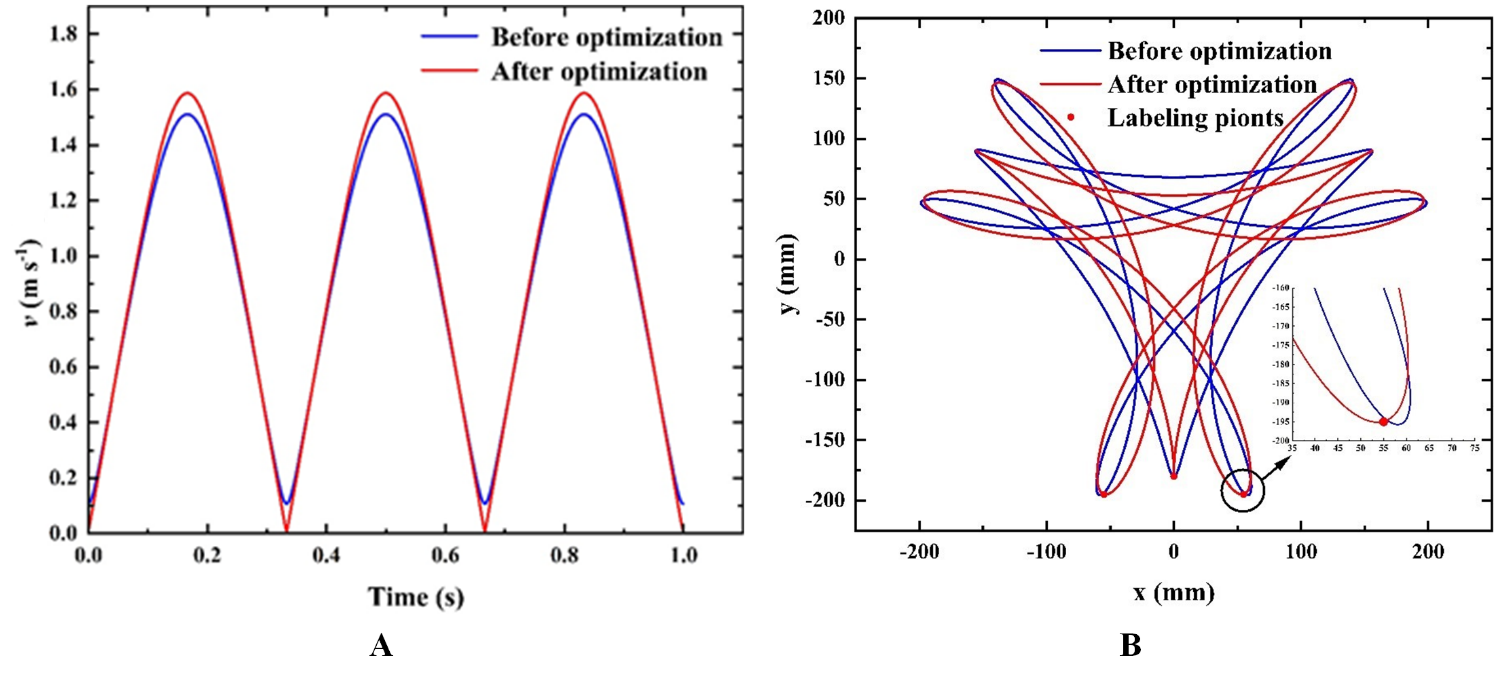


Supplementary Figure 7. (A) Optimized trajectory comparison; (B) Optimized velocity comparison


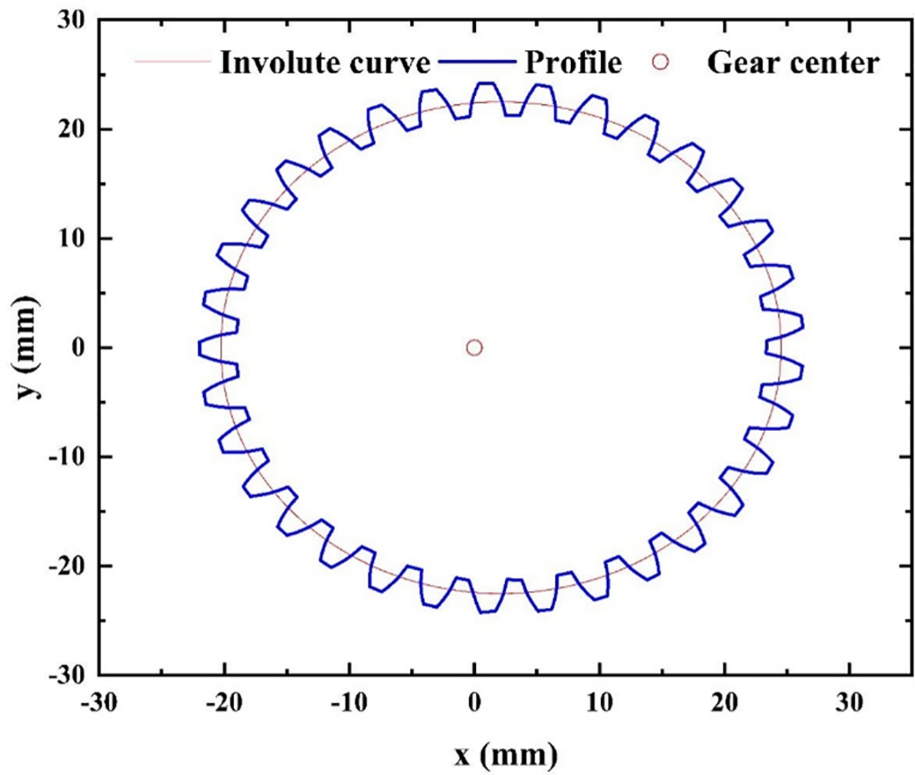


Supplementary Figure 8. Pitch curve and tooth profile of elliptical gear


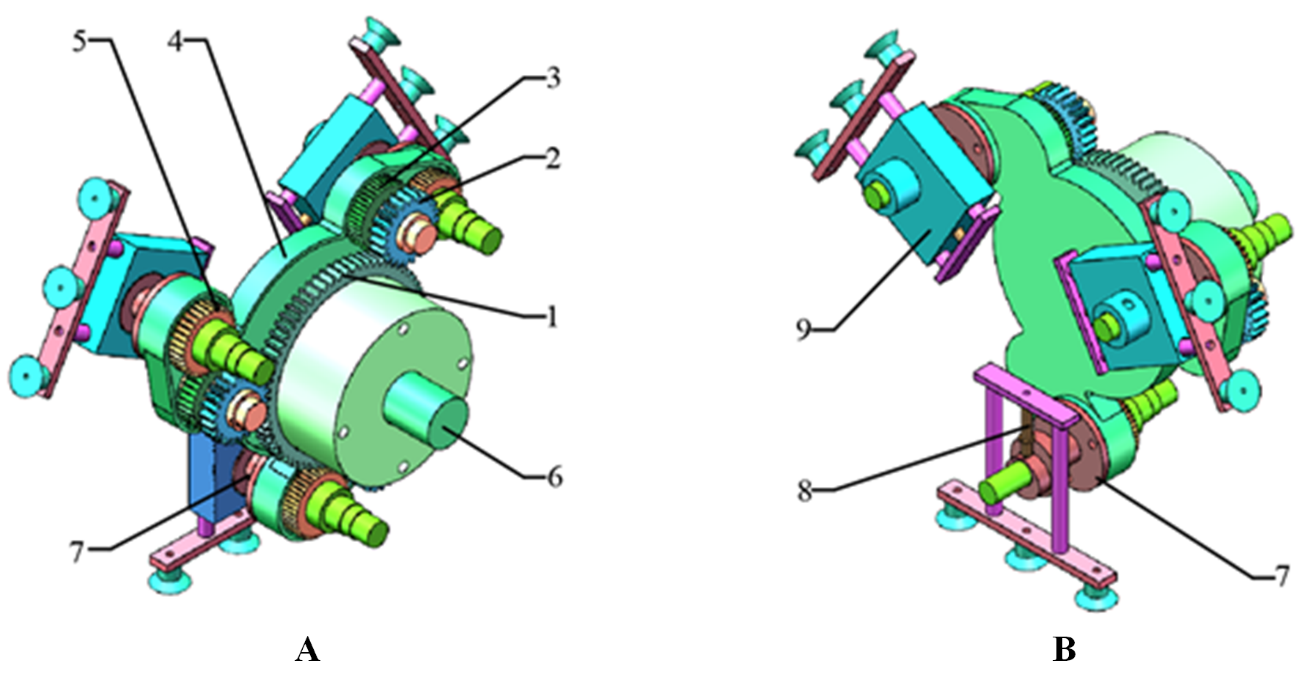


Supplementary Figure 9. Three-dimensional model of cam-elliptical gear combined labeling mechanism: (A). The back of mechanism, 1.sun gear 2.idler gear 3.elliptical idler gear 4.rotating bracket 5.elliptical planetary gear 6.fixed shaft 7.cam; (B). The front of mechanism, 8.cam lever, 9.sliding sleeve.


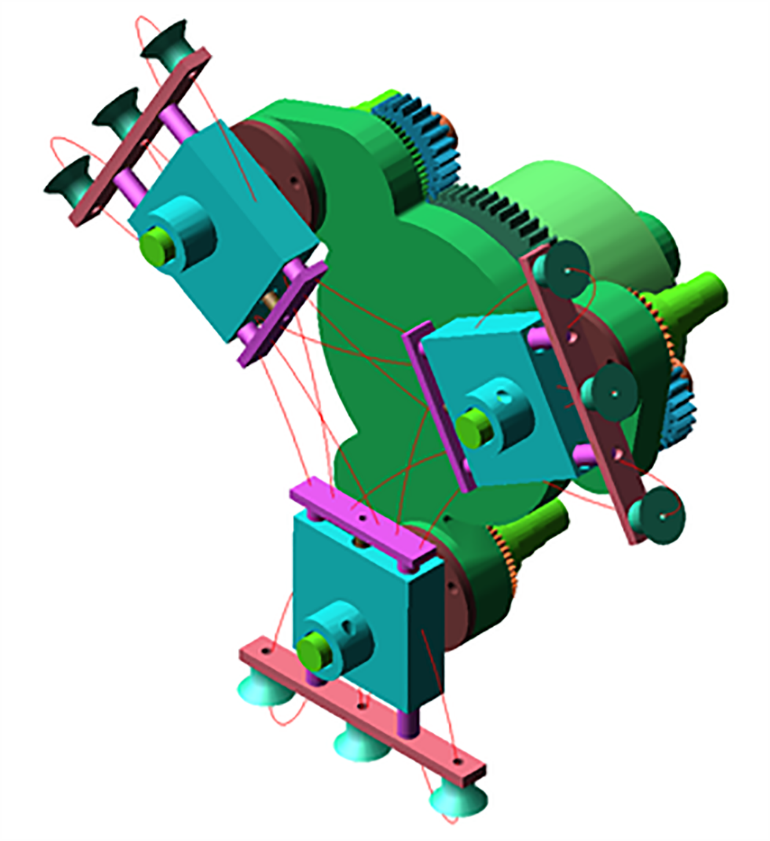


Supplementary Figure 10. Simulation trajectory


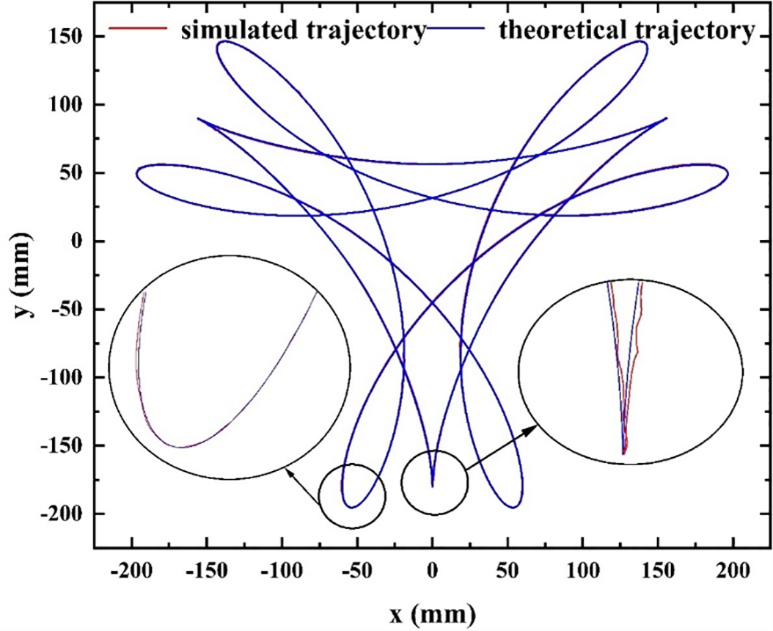


Supplementary Figure 11. Comparison between simulation trajectory and theoretical trajectory


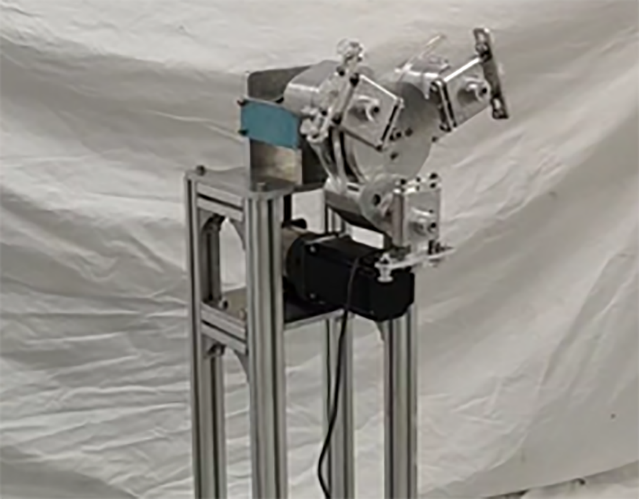


**Supplementary** **Figure 12**. Cam-Elliptical Gear Combination Vegetables Curved Surface Labeling Mechanism


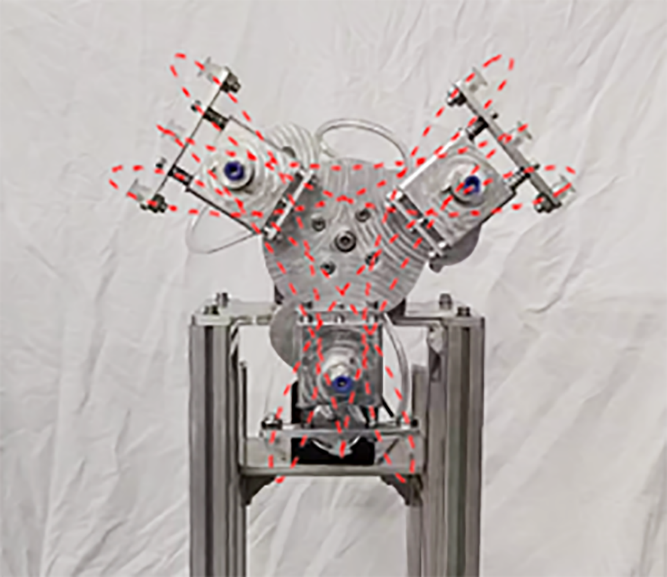


Supplementary Figure 13. Actual trajectory
